# Elasto-inertial focusing and particle migration in high aspect ratio microchannels for high-throughput separation

**DOI:** 10.1038/s41378-024-00724-2

**Published:** 2024-06-25

**Authors:** Selim Tanriverdi, Javier Cruz, Shahriar Habibi, Kasra Amini, Martim Costa, Fredrik Lundell, Gustaf Mårtensson, Luca Brandt, Outi Tammisola, Aman Russom

**Affiliations:** 1 Division of Nanobiotechnology, Department of Protein Science, Science for Life Laboratory, KTH Royal Institute of Technology, Solna, 171 65 Sweden; 2 Division of Microsystems Technology, Department of Materials Science and Engineering, Uppsala University, Uppsala, 752 37 Sweden; 3 FLOW and SeRC (Swedish e-Science Research Centre), Department of Engineering Mechanics, Royal Institute of Technology, Stockholm, SE 100 44 Sweden; 4 FLOW and Fluid Physics Laboratory, Department of Engineering Mechanics, Royal Institute of Technology, Stockholm, Sweden; 5 Wallenberg Wood Science Center, Royal Institute of Technology, Stockholm, SE 100 44 Sweden; 6 Department of Energy and Process Engineering, Norwegian University of Science and Technology (NTNU), Trondheim, Norway; 7 AIMES Center for the Advancement of Integrated Medical and Engineering Sciences at Karolinska Institutet and KTH Royal Institute of Technology, Stockholm, Sweden

**Keywords:** Microfluidics, Environmental, health and safety issues

## Abstract

The combination of flow elasticity and inertia has emerged as a viable tool for focusing and manipulating particles using microfluidics. Although there is considerable interest in the field of elasto-inertial microfluidics owing to its potential applications, research on particle focusing has been mostly limited to low Reynolds numbers (Re<1), and particle migration toward equilibrium positions has not been extensively examined. In this work, we thoroughly studied particle focusing on the dynamic range of flow rates and particle migration using straight microchannels with a single inlet high aspect ratio. We initially explored several parameters that had an impact on particle focusing, such as the particle size, channel dimensions, concentration of viscoelastic fluid, and flow rate. Our experimental work covered a wide range of dimensionless numbers (0.05 < Reynolds number < 85, 1.5 < Weissenberg number < 3800, 5 < Elasticity number < 470) using 3, 5, 7, and 10 µm particles. Our results showed that the particle size played a dominant role, and by tuning the parameters, particle focusing could be achieved at Reynolds numbers ranging from 0.2 (1 µL/min) to 85 (250 µL/min). Furthermore, we numerically and experimentally studied particle migration and reported differential particle migration for high-resolution separations of 5 µm, 7 µm and 10 µm particles in a sheathless flow at a throughput of 150 µL/min. Our work elucidates the complex particle transport in elasto-inertial flows and has great potential for the development of high-throughput and high-resolution particle separation for biomedical and environmental applications.

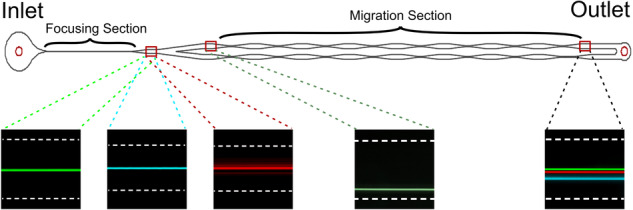

## Introduction

Microfluidics has been used in a wide range of research areas, such as biotechnology, medicine, chemistry, and the environment^1–4^, as it allows the control and manipulation of fluids in microchannels with high precision^5^. Therefore, this technology has been attractive for focusing, mixing, sorting, and separating particles suspended in various media^6^. Particle manipulation achieved using an external field such as electric^7^, magnetic^8^, or acoustic^9^ is referred to as active microfluidics. On the other hand, a system that relies only on features such as the channel geometry, fluid flow, particle size, and density is defined as passive microfluidics^10^. Deterministic lateral displacement (DLD)^11^, pinched flow fractionation (PFF)^12^, and inertial focusing^13^ are examples of such systems. Among the active and passive microfluidic techniques, inertial microfluidics is a robust, reliable, label-free, and high-throughput technique^14–16^.

Inertial focusing is the migration of randomly distributed particles toward equilibrium positions as they flow in microfluidic channels. The focusing phenomenon was initially observed in 1961 by Segre and Silberberg^17^. According to their observation, randomly distributed spherical particles at the inlet of a cylindrical pipe migrated and formed an annular ring that was located 0.6 times the pipe radius between the centerline and pipe wall at the outlet. Over the past two decades, other cross-sections, such as rectangles^18^, squares^19^ and triangles^20^, have been studied as microfluidic channels, and unique equilibrium positions have been found. These studies have shown that the balance between the shear-induced (*F*_*S*_) and wall-induced lift forces (*F*_*W*_) in Newtonian fluids defines the final equilibrium positions of the particles^6,21^. In addition to the microchannel cross-section, the Reynolds number (Re), which is a dimensionless number defined as the ratio of inertia over viscous time scales, plays a key role in particle migration. Unlike general microfluidic applications, where fluid inertia is negligible (Re ~ 0), inertial focusing occurs when fluid inertia is not negligible (Re > 1)^21,22^. Although interesting from a physics perspective, inertial focusing in straight channels with Newtonian fluids results in multiple equilibrium positions, thereby limiting its application. Single equilibrium positions can be achieved by adding curvature to the systems, but these systems work in a narrow bandwidth of conditions^23^, require high pressures when targeting micron and submicron particles and are more cumbersome to parallelize to increase the throughput than straight systems. On the other hand, the use of a non-Newtonian fluid, such as in elasto-inertial microfluidics, can easily achieve single-stream focusing of particles in straight microfluidic channels^24^.

While inertial microfluidics is based on the finite flow of Newtonian fluids, elasto-inertial microfluidics requires non-Newtonian fluids (viscoelastic fluids). The normal stress differences (*N*_*1*_ *– N*_*2*_) of a viscoelastic flow cause particles to experience an additional force called the elastic force (*F*_*E*_)^24,25^. The combination of elastic and inertial forces dictates the particle migration in the microchannels. Studies have shown that when inertial microfluidics is used in a rectangular microchannel, two focusing positions appear at the middle of the longer side of the channel. On the other hand, using the same cross-sectional channel with a viscoelastic fluid results in a single focusing position at the center of the microchannel^26^. This single focusing stream is caused by the particle migration toward the region with the lowest first normal stress difference, which is located in the middle of the microchannel^27^.

Due to the single-stream focusing of the particles in a straight microfluidic channel, the use of elasto-inertial microfluidics is convenient for particle focusing, concentration and size-based particle separation. However, the mechanisms behind the particle migration in viscoelastic fluids are not completely understood and are still being investigated. Previous studies have concentrated on understanding the single-stream focusing behavior using simulation models^26,28^. Although these models appear promising for explaining single-stream focusing, they still need to be validated by experimental results to become reliable predictive tools. Practical applications using microchannels with rectangular cross‐sections have been limited to relatively low Re (0.03–0.4)^29,30^. Recent studies using microchannels with a circular cross‐section have shown that it is possible to focus particles using high Re (~ 100), similar to inertial microfluidics^31,32^. Furthermore, using spiral devices, the particles at the outer channel wall can be focused, and the particles at relatively high Re (5–50) can be separated^33,34^. In addition, the particle separation applications in the previous studies considered two inlets where one inlet carried the sample flow and the second inlet carried a sheath flow^33–37^. The utilization of the sheath flow decreased the overall throughput. To overcome these issues, we present a microfluidic channel with a single inlet, which initially focuses all particles and then separates them by size by exploiting the difference in their migration speed.

In this study, we experimentally demonstrate particle focusing in high aspect ratio (AR=height/width) straight microchannels using 3, 5, and 10 µm particles with viscoelastic fluids at four different concentrations of polyethylene oxide (PEO), ranging from dilute to semi-dilute regimes. In addition to different particle sizes and viscosities, the effect of the channel geometry on particle focusing is examined by altering the channel width and height while maintaining a high aspect ratio.

The presented experimental data provide insights into several aspects of particle focusing in non-Newtonian fluids. The particle size emerges as the dominant parameter for particle focusing. Using the novel devices, we prefocus the particles and then split the channel into two parts to force the particles to reach a position near the channel wall and observe their migration toward the middle of the channel. The obtained data show the trajectories, as well as their dependency on the particle size, fluid flow, viscoelastic concentration, and channel geometry. Furthermore, we report a numerical study of the size-based particle migration in viscoelastic fluids. Finally, we demonstrate that size-based separation can be achieved by exploiting the different migration speeds toward the central line.

### Theoretical background

The flow in a microchannel can be described as either laminar or turbulent^38^. The Reynolds number (Re), a dimensionless number, defines the flow characteristics in a microfluidic channel. It is the ratio between the inertial and viscous time scales and can be expressed as follows^39^:

1 $${\mathrm{Re}}=\frac{\rho {U}_{m}L}{\mu },$$

where *ρ* is the density of the fluid, *U*_*m*_ is the maximum flow velocity, *L* is the characteristic channel length and *µ* is the viscosity. When Re is <2300, the flow is defined as laminar flow, while if Re is >2300, the flow is turbulent. In inertial and elasto-inertial microfluidics, the flow is always in the laminar regime: the fluid flows in parallel layers without mixing, unlike in a turbulent flow^38,40^. In conventional microfluidics, Re is <1, while in inertial microfluidics, Re is typically >1 and is still well below 1000. In this regime, the flow is laminar, but the inertia is not negligible. At finite Reynolds numbers, particle focusing in a straight microchannel occurs when the two forces balance each other, i.e., shear-induced lift force (*F*_*S*_) toward the wall and wall-induced lift force (*F*_*W*_) away from the wall^41^. Initially, randomly distributed particles are pushed away from the center of the channel toward the wall due to the shear-induced lift force that arises from the characteristic Poiseuille flow^42^. As particles approach the wall, a counterforce, the wall-induced lift force, develops and pushes particles toward the center of the channel. The resultant force is called the lift force (*F*_*L*_) and is defined as follows^41^:

2 $${F}_{L}={C}_{L}\frac{\rho {U}_{m}^{2}{a}^{4}}{{D}_{h}^{2}},$$

where *C*_*L*_ is the lift coefficient, *a* is the particle size and *D*_*h*_ is the channel hydraulic diameter and is calculated as $2{wh}/(w+h)$, where *w* is the width and *h* is the height of the microchannel. The ratio of the particle size to the hydraulic diameter provides the blockage ratio (*β*)^43^. This ratio should be >0.06 to focus the particles according to a previous study^33^.

In addition to the lift force, an elastic force occurs when the fluid is non-Newtonian and plays a role during particle migration in elasto-inertial microfluidics. This elastic force is expressed as follows^44^:

3 $${F}_{E}={C}_{e}{a}^{3}\nabla {N}_{1},$$

where *C*_*e*_ is the elastic coefficient and *N*_*1*_ is the first normal stress difference.

Particles in a microfluidic channel experience *F*_*L*_ and *F*_*E*_ and obtain their equilibrium positions when all these forces balance each other. To quantitatively characterize a viscoelastic flow, several dimensionless numbers, such as the Weissenberg number (Wi) and the elasticity number (El), are utilized. The Weissenberg number is the ratio of the elastic force to the viscous force and is defined as^45^ ${Wi}=2\lambda Q/h{w}^{2}$, where *λ* is the characteristic relaxation time of the fluid and *Q* is the volumetric flow rate. The elasticity number describes the ratio of the elastic force to the inertial force and is defined as^46^ ${El}={Wi}/\mathrm{Re}$, or^46^ ${El}=\lambda \mu (w+h)/\rho {w}^{2}h$.

## Results and discussion

### Particle focusing

**Fig. 1:**
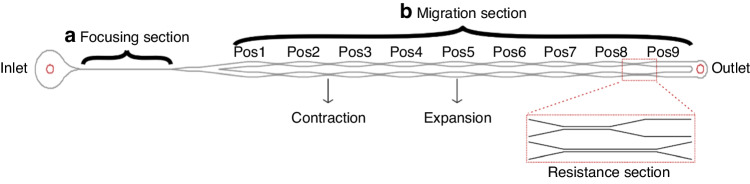
Design of the microfluidic chip. **a** Focusing section - the inlet is followed by a straight focusing section. **b** Migration section - the main channel is split in two parallel channels where bottom channel has slightly higher resistance as highlighted in the box. This resistance causes particles to follow upper channel that allows to observe migration

Elasto-inertial particle focusing occurs due to the complex relationship between *F*_*E*_ and *F*_*L*_ and is affected by various parameters, such as the particle size, channel geometry, and polymer concentration in the fluid, as stated in the introduction. Here, we experimentally determine the impact of each of these parameters on the particle focusing over a wide range of flow rates. Experimentally, the data were analyzed at the end of the focusing section of the microfluidic channels (Fig. 1a). Detailed information regarding the microfluidic channels can be found in the Materials and Methods section. Below, each of the parameters affecting the particle focusing are described.

#### Effect of the particle size

**Fig. 2:**
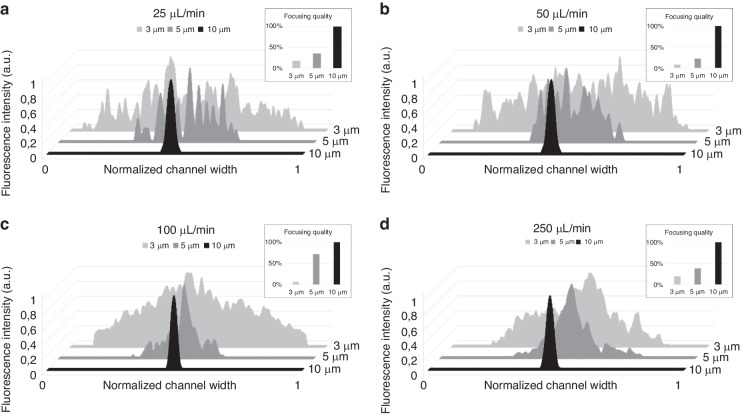
Effect of the particle size on focusing at different flow rates. Fluorescence intensity graphs of 3, 5 and 10 µm particles in microchannel (*h* = 60 µm, *w* = 40 µm) at a PEO concentration of 4000 ppm for (**a**) 25 µL/min, (**b**) 50 µL/min, (**c**) 100 µL/min and (**d**) 250 µL/min. Focusing qualities of particles in each case are shown in the boxes

To study the effect of particle size on focusing, 3, 5 and 10 µm particles were used in a microfluidic channel (*h* = 60 µm, *w* = 40 µm) with a fixed PEO concentration of 4000 ppm over four different flow rates (25, 50, 100 and 250 µL/min). The normalized fluorescence intensities across the channel width are shown in Fig. 2. In addition to the intensity graph, the focusing quality of the particles is calculated and shown in the corresponding figures. Regardless of the flow rate, the largest particle (10 µm) shows the highest focusing quality compared to the 3 and 5 µm particles, while the smallest particle (3 µm) shows the lowest focusing behavior at any recorded flow rate. Increasing the flow rate 10 times from 25 µL/min (Re = 1.03, Wi = 149.52) to 250 µL/min (Re = 10.30, Wi = 1495.15) did not have any effect on the focusing of the 10 µm particles since they maintained single-line focusing at the middle of the channel. Although the 3 µm particles showed a slight improvement with respect to the focusing quality at 250 µL/min, the particles could not achieve single-line focusing at any flow rate, reaching <25% focusing quality at best. On the other hand, the focusing quality of 5 µm particles was significantly enhanced at a flow rate of 100 µL/min, reaching almost single-line focusing. However, a further increase in the flow rate to 250 µL/min caused a decrease in the focusing quality, while the 10 µm particles were fully focused at this high flow rate. These results indicate that the particle size plays a major role in single-line focusing in viscoelastic fluids. Further investigation of the particle focusing was performed by altering the channel width and PEO concentration while keeping the height of the channel constant (see Supplementary Material Fig. S1). For any experimental condition, we observe that focusing improves as the particle size increases. The dominant role of the particle size can be attributed to the cubic scaling of the elastic force (*F*_*E*_ ~ a^3^)^47^.

Moreover, when Re < 1 and the PEO concentration is in the dilute regime (c < 858 ppm), the particles tend to be located in the corners of the channel, the regions of lower stress^48^, and this behavior is more pronounced for smaller particles (β < 0.1). Under the same flow conditions, as the particle size increases, the probability of observing particles in the corners decreases (see Supplementary Material Fig. S2). Moreover, increasing the flow rate (thus increasing the Reynolds number) confines low stress regions in viscoelastic fluid^36^, and a corresponding increase in the elastic force drives particles away from the channel corner. This migration is faster for larger particles (β > 0.2), leading to focusing at the channel center earlier than for smaller particles.

#### Effect of the channel geometry

**Fig. 3:**
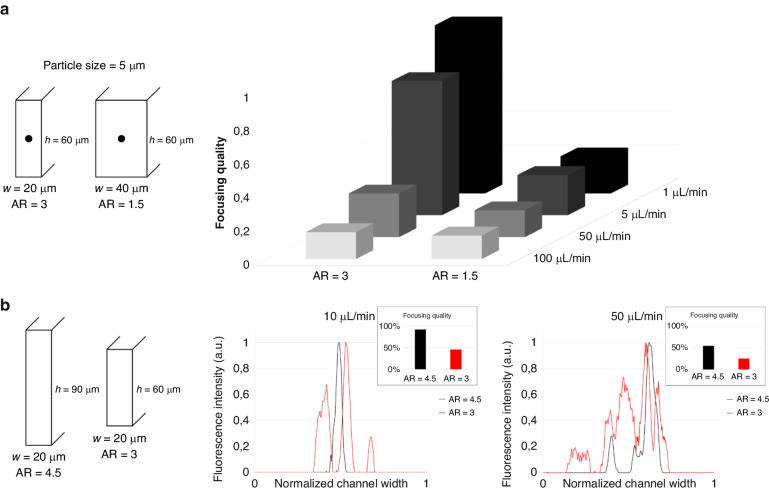
Effect of the channel geometry on particle focusing. Cross-sections of each case are shown on the left side. **a** Effect of the channel width- Focusing quality of the 5 µm particles in the microchannels with two different width (*w* = 20,40 µm, *h* = 60 µm) **b** Effect of the channel height- Fluorescence intensity graphs and focusing quality of the 5 µm particles in the microchannels (*h* = 90,60 µm, *w* = 20 µm)

To understand the effect of channel width on particle focusing in high aspect ratio microchannels, we used two different widths (20 and 40 µm) while keeping the channel height at 60 µm (AR = 3 and 1.5), as illustrated in Fig. 3a; here, we also display the focusing quality of the 5 µm particles in both microfluidic channels at flow rates of 1, 5, 50 and 100 µL/min (PEO = 1000 ppm). The experimental data show that the focusing quality of 5 µm particles improves in each case as the channel width decreases (*w* = 20 µm), and a significant decrease in the focusing quality is observed when the channel width increases (*w* = 40 µm). This behavior is caused by the inverse proportionality between the elastic forces and channel width (*F*_*E*_~1/w^3^)^49,50^. Although a decrease in the channel width increases the focusing quality, the enhancement ratio varies with the change in the flow rate. The greatest improvement was observed when the flow rate was 5 µL/min, indicating that the effect of channel width was more significant at lower flow rates.

Increasing the flow rate from 1 µL/min to 100 µL/min reduced the focusing quality for each dataset (Fig. 3a). The reduction in the focusing quality at 50 and 100 µL/min is caused by the increased effect of inertia, which pushes the particles away from the channel center^48,51^. Moreover, the decrease in the focusing quality was more significant for smaller channels (*w* = 20 µm). This was likely the result of a stronger *N*_*2*_-induced secondary flow in smaller channels^52^. From an application viewpoint, one aim is to focus particles at high flow rates to increase the throughput. Therefore, we tested the effect of the channel depth while maintaining the channel width. Figure 3b shows the focusing quality of 5 µm particles in the channels with aspect ratios of 4.5 (*h* = 90 µm*, w* = 20 µm) and 3 (*h* = 60 µm, *w* = 20 µm) at flow rates of 10 µL/min and 50 µL/min, respectively. In the channel with a lower aspect ratio, 80% of the particles were focused at 5 µL/min. However, increasing the flow rate to 10 µL/min and 50 µL/min reduced the focusing quality to 46% and 26%, respectively. On the other hand, our results showed that when the channel aspect ratio was increased to 4.5, particles reached a focusing quality of 92% for 10 µL/min and 55% for 50 µL/min. To further investigate this aspect, we compared the focusing quality of 10 µm particles in channels with aspect ratios of 3 (*h* = 120 µm, *w* = 40 µm) and 1.5 (*h* = 60 µm, *w* = 40 µm). The focusing quality was 40% and 17% at 50 µL/min (0.1 wt. % PEO) and 100 µL/min (0.05 wt.% PEO), respectively, in the smaller channel. When doubling the aspect ratio, the particles were completely focused at a flow rate of 50 µL/min (0.1 wt. % PEO), and the focusing quality increased from 17% to 80% at 100 µL/min (0.05 wt. % PEO) (Supplementary Material Fig. S3). As expected, our findings indicated that in high aspect ratio channels, increasing the channel height enabled the focusing of the particles at higher flow rates when using viscoelastic fluids. In addition to the effect of channel width and height, we also investigated the effect of channel length on particle focusing. Our results showed that a longer channel length resulted in better focusing (see Supplementary Material Fig. S4).

#### Effect of the PEO concentration

The variation in the PEO concentration directly affects the elastic (*F*_*E*_ ~ *λ*)^47^ and viscous forces (Re ~ 1/µ)^22^ acting on a particle flowing in the channel. Therefore, particle focusing is strongly influenced by the concentration of PEO. To analyze the effects of varying PEO concentrations on the elasticity and viscosity of PEO, four different solutions of PEO were prepared (500, 1000, 2000 and 4000 ppm). The rheological properties of these solutions are shown in Supplementary Material Table S1.

**Fig. 4:**
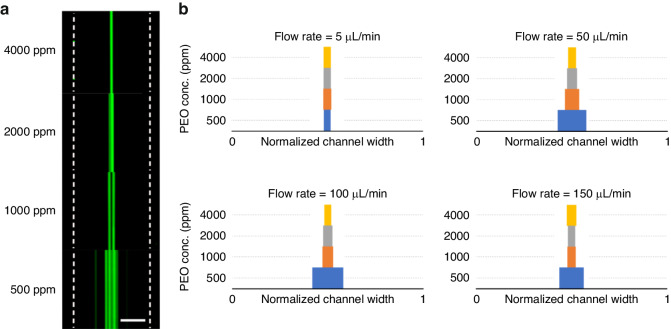
Effect of PEO concentration on focusing. **a** Experimental data of focusing of 10 µm particles at different PEO concentrations (Flow rate = 50 µL/min). The fluorescence signals were recorded at the end of the focusing section. The dashed lines are the channel walls. Scale bar: 100 µm **b** Focusing bandwidths (FWHM) over normalized channel width of 10 µm particles for four different PEO concentrations from 5 – 150 µL/min. Increasing the PEO concentration improves focusing quality more significantly at higher flow rates

Fluorescence imaging of the 10 µm particles at different PEO concentrations shows the effect of the elasticity on the particle focusing at 50 µL/min (see Fig. 4a). Moreover, the focusing qualities of 10 µm particles in a microchannel (*h* = 60 µm, *w* = 40 µm) with flow rates varying from 5 µL/min to 150 µL/min for all PEO concentrations are shown in Fig. 4b. At 5 µL/min in 500 ppm PEO solution, the particles were fully focused, and an increase in the PEO concentration maintained the focusing quality. However, increasing the flow rate to 50 µL/min caused a significant decrease in the focusing quality in the solution with 500 ppm PEO due to the stronger inertial and elastic forces generated by the higher flow rate^48^. Increasing the PEO concentration to 1000, 2000, and 4000 ppm resulted in continuous improvements until the particles were fully focused at the highest PEO concentration. A similar trend was observed for a flow rate of 100 µL/min. These improvements in particle focusing could be attributed to the increase in the elasticity of the channels at higher PEO concentrations. Similar results were obtained for the 5 µm particles at relatively high flow rates (100–150 µL/min) (Supplementary Material Fig. S5 in the Supplementary Material section). These results indicate that particle focusing at high flow rates (>50 µL/min) can be achieved by increasing the viscoelasticity of the fluid.

As shown above, particle focusing depends on various parameters (particle size, channel geometry, viscoelasticity), and focusing can be achieved by tuning these parameters at low and high flow rates. Fully focused particles are essential for our high-throughput and high-resolution particle separation strategy, which is described in detail below.

### Particle migration

Knowledge of the migration velocity is fundamental for sorting particles by size, as described below, and for designing devices with the desired throughput and reasonable dimensions. To this end, we used the migration section of the microfluidic channels (see Fig. 1b) and followed the particle trajectory throughout the microfluidic channel, as well as the final focusing positions close to the outlet. The particles are first focused, and then the channel is split into two parts to track the particle migration from the sidewall toward the center. Each data point was recorded in the expansion section of the microchannels.

#### Numerical study of particle migration

**Fig. 5:**
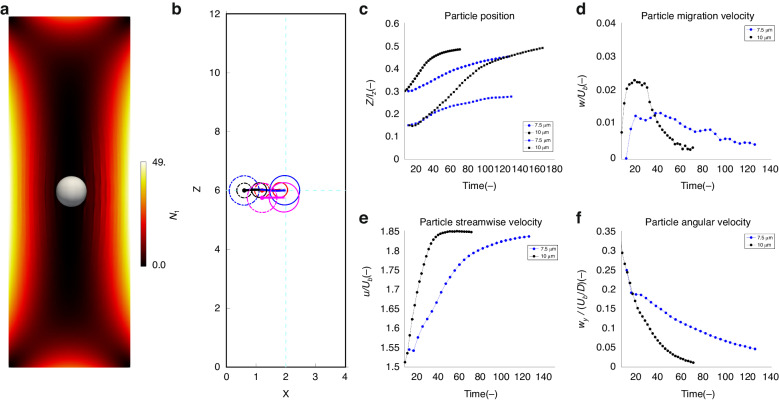
Numerical study of particle migration. **a** First normal stress difference (*N*_1_) across the cross-section of the channel. The particle attains its equilibrium position at the center of the channel where *N*_1_ = 0. **b** Migration of 7.5 and 10 µm particles from two different positions near the wall toward the channel centerline. The dashed lines represent the starting positions and the solid lines represent the final measured positions. **c** Particle spanwise position. Two different starting positions (0.15 W and 0.3 W) are shown. **d** Particle migration velocity. **e** Particle streamwise velocity. **f** Particle angular velocity

To understand the transient dynamics behind the migration of particles toward the centerline, we need to consider the competition between the elastic and inertial forces in the fluid. The particles are pushed away from the centerline by the shear gradient lift force (*F*_*S*_) and toward the centerline by the elastic forces. Our simulations, with values of Wi = 13.55 and Re = 6.65, reveal that the elastic forces are more influential than the inertial forces in our setup. This results in the particles being mainly directed toward the center of the channel by the prevailing elastic forces. To clarify, the distribution of the first normal stress difference of the viscoelastic fluid, *N*_1_ = *τ*_*xx*_ − *τ*_*yy*_, across the channel cross-section is depicted in Fig. 5. Note that the first normal stress difference indicates tension in the streamline direction. The streamlines surround the particle and exert a lateral hooping thrust on each side of the particle, causing it to move to the side of the particle with the lowest first normal stress difference. The simulations show that *N*_1_ is the greatest near the four walls of the channel, while it is significantly lower close to the channel center and corners. As a result, the gradients of the first normal stress difference tend to push the particles toward the center of the channel where the elastic stresses are minimal.

Although the migration is consistently significant along the short channel (*Z*-axis), migration along the longer edge (*Y*-axis in Fig. 5) is not as pronounced because the region with the lowest *N*_1_ values at the center of the channel has a rectangular shape. When the elastic effects are substantial, the particles are inclined to move toward this central rectangular region, as shown in Fig. 5b. As a result, the migration along the long side is slower, and several locations in the midplane cutting along the Y-direction can potentially act as an equilibrium position.

To further understand the dynamics of particle migration, we investigated the influence of particle size on migration. Specifically, we numerically simulate particles with diameters of 10 µm and 7.5 µm under constant flow rate and fluid rheological properties. The migration behavior of these particles is illustrated in Fig. 5c–f. The results in panel (c) indicate that both particles migrate toward the channel center at (*Z*/*l*_*z*_ = 0.5) because the elastic forces are predominant over the inertial forces. Notably, the particle with a smaller diameter (7.5 µm) reaches the final steady state later than its larger counterpart (10 µm). The larger particle has already reached the final equilibrium position, while the smaller particle continues its gradual migration toward the channel center. The migration velocities of the particles are compared in Fig. 5d: a larger particle exhibits a higher migration velocity than a smaller particle. This difference can be attributed to the elastic effects arising from the imbalance in the distribution of *N*_1_ over the particle surface. This is expressed by *F*_E_ ∝ *a*^3^∇(*N*_1_), where the cubic dependence results from the product between the surface area over which the stresses act and the gradients in the normal stresses proportional to the particle size. Consequently, the larger particle experiences a more pronounced elastic force, accelerating its migration toward the centerline of the channel. In addition, for both smaller and larger particles, the migration velocity decreases as the particles approach the channel center. This is due to the decreasing gradient of the first normal stress difference as the particles approach the center of the channel (Fig. 5a), causing a reduction in elastic forces. In panel (e), the streamlines of the particles normalized by the bulk velocity (*u*/*U*) are presented; here, an increase in the particle streamwise velocity is observed as the fluid dynamics cause the particle to align with the prevailing flow. As the particles approach a stable state, their streamwise velocity becomes constant, closely aligning with the velocity of the fluid at the equilibrium point (at the center of the microchannel). Finally, in panel (f), we present the angular velocity of the particle around the *y*-axis. Evidently, the angular velocities decrease and eventually approach zero. This behavior can again be attributed to the diminishing gradient in both *N*_1_ and the vanishing shear rate as particles move closer to the center of the channel^53^. Since this force governs the particle rotation, the angular velocity gradually decreases and eventually approaches zero.

#### Experimental study of particle migration

**Fig. 6:**
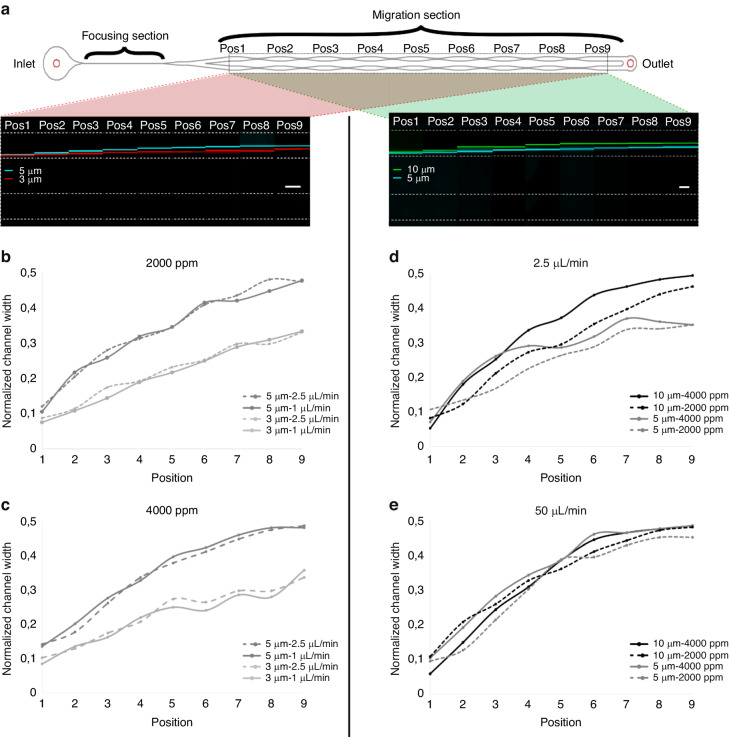
Particle migration trajectories. **a** Schematic of a microfluidic chip with the focusing and migration section. Highlighted boxes (left, *w* = 20 µm and right, *w* = 40 µm) show representative experimental results of the particle migration from position 1 to 9 at a PEO concentration of 4000 ppm. White dashed lines are the channel walls. Scale bars: 100 µm. **b**, **c** Trajectories of 3 µm and 5 µm particles at PEO concentrations of (**b**) 2000 ppm and (**c**) 4000 ppm at flow rates of 1 µL/min and 2.5 µL/min. The channel AR = 3 (*w* = 20 µm). **d**, **e** Trajectories of 5 µm and 10 µm particles at PEO concentrations of 2000 and 4000 ppm at flow rate of (**d**) 2.5 µL/min and (**e**) 50 µL/min. The channel AR = 1.5 (*w* = 40 µm)

Following the numerical study, we thoroughly investigated elasto-inertial particle migration in high aspect ratio straight microchannels. To this end, after the straight portion of the microfluidic device where focusing occurs, we split the microchannel into the two parallel straight channels, and one of these channels is slightly longer (Table 1). This causes a higher resistance in the longer channel and drives the particles toward the lower resistance side, near the channel wall. In this way, we achieve controlled positioning of the particles near the channel wall for a more accurate analysis of the migration. As introduced above (Fig. 1), parallel channels were designed with expansion and contraction units to observe particle migration to the center. Figure 6a shows the fluorescence data from two representative experimental results using 3 µm and 5 µm particles (left side) and 5 µm and 10 µm particles (right side). The results showed that larger particles migrated toward the channel center earlier than smaller particles in both cases. Notably, as the particles approach the channel center, the migration speed decreased, in agreement with the numerical study above. The variation in the migration speed for particles of different sizes could be utilized for separation. Since the larger particle slowed down and eventually stopped migrating at the center, small particles reduced this distance by continuing their migration if the channel provides sufficient length. Figure 6b, c shows the effect of the PEO concentration on particle migration for two different flow rates. As the PEO concentration increases, the larger (5 µm) particles migrate faster and reach the center earlier. This result is indicative of the relatively dominant viscoelastic force (*F*_*E*_) over the lift force (*F*_*L*_). For instance, the dimensionless numbers for 2000 ppm at a flow rate of 2.5 µL/min are as follows: Re = 0.45 and Wi = 38.11, while for 4000 ppm, the corresponding Re = 0.13 and Wi = 59.81. These results show that while the influence of *F*_*L*_ is moderate, the influence of *F*_*E*_ is the dominant force causing the movement of the particles across the channel toward the center. As shown in Fig. 6d, the trend is similar for the microchannel with AR = 1.5. The 10 µm particles reach the channel center earlier at a PEO concentration of 4000 ppm than at 2000 ppm (corresponding to Re = 0.35 and 0.1 and Wi = 9.53 and 14.95). At these relatively low flow rates, a strict size-based differential particle migration occurs (Fig. 6b–d). As the flow rate increases, the size dependence of the particle migration is reduced. At a flow rate of 50 µL/min (for flow through the channel AR = 1.5), both 10 µm and 5 µm particles migrate at similar speeds (Fig. 6e). Most notably, for a PEO concentration of 4000 ppm, the migration path between the 10 µm and 5 µm particles is difficult to differentiate, resulting in a practically impossible separation. This effect is presumably due to the dominant *F*_*E*_ over *F*_*L*_ (Re = 2.06, Wi = 299.03) at these high PEO concentrations. Overall, these results document the size dependence of particle migration at optimized flow rates and can be exploited for high-resolution separation.

**Table 1:** Dimensions of the microfluidic channels

Channel Width	Focusing Length	Contraction Length	Expansion Width	Resistance Length
20 µm	4 mm	0.25 mm	150 µm	0.75 mm
40 µm	8 mm	0.5 mm	300 µm	1.5 mm

To gain more insight into the complex relationship between the forces affecting particle migration toward the channel center, we analyzed a large amount of experimental data at the outlets (position 9 in Fig. 6a) using particles of different sizes, PEO concentrations, channel aspect ratios and flow rates. Our observations are summarized in the Supplementary Material (Supplementary Material Fig. S6). Briefly, to analyze the final particle positions at the outlet, we denoted *W*_*n*_ as the normalized channel width, from 0 at the inner wall to 1 at the outer wall of the channel. Our analysis shows that particles are primarily positioned in two regions: the channel center and a region from 0.28 to 0.35*W*_*n*_. We observe that this trend is more significant at higher flow rates (>50 µL/min), indicating the effect of secondary flow (see Supplementary Material Fig. S6). Particles that are sufficiently large and under the influence of a dominant *F*_*E*_ are expected at the equilibrium position at the center. However, the second focusing region (0.28 to 0.35*W*_*n*_) is distinguished with notable results. The secondary flow present at the high flow rates potentially competes with the dominant *F*_*E*,_ pushing particles away from the channel center. In these cases, the combined effect of *F*_*L*_ and secondary forces would balance the effect of the *F*_*E*_. Note that we can observe this phenomenon since all particles are forced to start from the inner wall and migrate toward the channel center due to the channel design, which allows prefocusing of the particles before splitting. However, outside the focus of the current work, a more detailed study is needed to determine the effect of the secondary flows due to the viscoelastic property of the medium and this interaction of the *F*_*E*_ and *F*_*L*_ in the focused particles.

### Differential particle migration for high-throughput separation

**Fig. 7:**
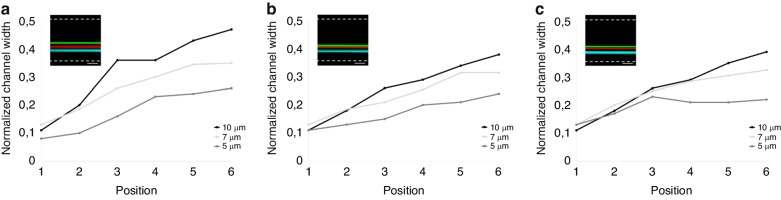
Particle trajectories from position 1 to 6 with experimental images of 5 (cyan), 7 (red), and 10 (green) µm particles at position 6. Separation of 5, 7, and 10 µm particles at a fixed PEO concentration (1000 ppm) is shown at flow rates (**a**) 50 µL/min, (**b**) 100 µL/min and (**c**) 150 µL/min. (*w* = 40 µm, *h* = 120 µm) Dashed lines are the channel walls. Scale bars: 100 µm

For particle separation, high sample throughput without compromising the separation resolution is desirable. As described above, pre-aligning the particles without a sheath flow is attractive and enables the strict size-based particle migration toward the channel center after splitting. From the numerical and experimental results, we observe that particles start to slow down in migration when they approach the channel center. Hence, a strategy can potentially be built based on straight channels that allows the larger particles to migrate to reach the channel center while not providing sufficient length/time for smaller particles to reach the channel center. Experimentally, for channel AR = 3, the largest difference is observed just before the larger particles start to slow down (see position 6 in Fig. 6b, c). For high-throughput separation applications, we fabricated a deeper device microchannel (*w* = 40 µm, *h* = 120 µm, keeping the AR = 3) and analyzed the particles at position 6. As described above, the PEO concentration is another important parameter to be optimized for particle separation at high flow rates. As shown in Fig. 7, at a PEO concentration of 1000 ppm, we demonstrated high-resolution separation of 5, 7, and 10 µm particles at high flow rates (50, 100, and 150 µL/min). As expected, a lower flow rate (50 µL/min) resulted in a larger separation distance, and we also observed that 7 µm particles occupied an intermediate position between 5 and 10 µm, confirming size-based differential migration. At slightly different positions, we observed the particles entering the second section of the channel at ~0.1 W (close to the inner wall). Independent of the initial position, the larger 10 µm particles migrated faster than the smaller particles toward the channel center. Moreover, high throughput was achieved by increasing the channel depth to 120 µm. Note that the aspect ratio (AR = 3) was maintained constant by keeping the width at 40 µm. By designing collection outlets, the separation of particles at high resolution should be straightforward.

Straight microfluidic channels have been used in the past for particle separation based on size^36,54,55^. However, these studies have used two inlets: one containing the fluid with particles and the other containing only fluid as the sheath flow^37,55,56^. In these configurations, the sheath flow serves to initially define the particle position in the channel and moves the particles to the chosen side for further migration. Although these designs effectively work for particle separation, the main particle-laden flow is often at very low flow rates, and consequently, the sheath flow causes a dilution of the solution, significantly lowering the overall sample throughput. Spiral devices are attractive for focusing and separating the particles. The combination of the Dean force that arises from a curvature and elasto-inertial flow causes particles to focus at the outer wall of the microchannel^57^, which allows size-based particle separation^58,59^. However, these spiral geometries also suffer from a decrease in throughput due to the use of the sheath flow. In addition, these systems require additional control systems to operate due to the multiple inlets and more complicated designs to initially define the particle positions to enable size-based manipulation. Here, we demonstrate that by first pre-aligning the particles, the difference in the migration speed can be utilized for separating particles at high throughput and high resolution based on their sizes.

## Conclusions

In this study, we report a numerical and comprehensive experimental investigation of the particle focusing and migration in elasto-inertial flows through straight high aspect ratio microchannels. The data show the effects of particle size, channel geometry, flow rate, and the concentration of polymer additives on the particle focusing for Reynolds numbers, Re, in the range of 0.05–85, Weissenberg number, Wi, between 1.55 and 3800 and a large range of elasticity numbers (=Wi/Re), El, in the range of 5.75–462. For the first time, this range of Wi and El numbers is experimentally investigated, and based on the experimental data, the elasticity and inertia can be used to focus particles by tuning various parameters. Among these parameters, the particle size plays a dominant role in particle focusing. In addition to focusing, we report particle migration trajectories from the near wall toward the channel center and show the effect of the particle size, flow rate, and PEO concentration on this migration. Particles migrate faster when they are near the channel wall, and their migration becomes slower as the particles approach the center of the channel. Exploiting this result, the differences in the migration speeds can be utilized for separating particles at high throughput and high resolution. As a proof of principle, we demonstrated successful separation of 5 µm, 7 µm and 10 µm particles at a throughput of 150 µL/min using a single inlet channel without sheath flow to pre-align the particles.

## Materials and methods

### Device fabrication

Microfluidic devices were designed using AutoCAD software (Autodesk), and the master mold was prepared with SUEX (dry SU-8 film) on a silicon wafer following a standard photolithography process^60^. To fabricate the PDMS (polydimethylsiloxane) chips, SYLGARD™ 184 elastomer (Dowsil, Sweden) was mixed with the curing agent at a ratio of 10:1, and the final mixture was poured over the master mold, degassed in a desiccator and baked at 65 °C for 6 h. After the curing process, the PDMS was peeled off, the individual devices were cut, and the inlet and outlet holes were punched using a puncher with a hole size of 0.75 mm. The resulting PDMS device and a microscope glass slide were exposed to the oxygen plasma to activate the surfaces for bonding. After bonding the PDMS device on the glass substrate, the chip was post-baked at 120 °C for 15 min to improve sealing.

### Design of the microfluidic channels

To study the effect of the channel width on particle focusing and migration, straight microfluidic channels with two different widths (20 µm and 40 µm) and constant height (60 µm) were designed. Both microfluidic chips consisted of a first section for focusing all particles in the middle and a second section where the already focused particles were quickly displaced toward the inner channel wall and were observed as they migrated back to the center. In the second section, the trajectories could be observed, and their dependencies on the particle size, channel width, flow rate, and concentration of elasticity were examined. The first section was a straight microchannel (Fig. 1a), and in the second section, the channel was split into two branches, with one branch receiving 55% of the fluid, while the other branch received 45% of the fluid (Fig. 1b). Prefocused particles entered the second section very close to the wall of the channel that received 55% of the flow and started migrating toward the center of the new channel. To properly observe the position of the particles, this second focusing channel was segmented into 8 parts with an expanded peak and high resolution at 8 different points of the trajectory. The 40 µm wide channel was designed with an 8 mm long particle focusing section followed by a second section with 8 segments of 500 µm. The 20 µm chip was designed with a 4 mm long focusing section and 8 segments of 250 µm (Table 1).

### Sample preparation

Non-Newtonian fluid was prepared by dissolving polyethylene oxide (PEO) powder, which is used as an elasticity enhancer, in deionized water. PEO powder (M_w_ = 2 × 10^6 ^g/mol, Sigma Aldrich) was added to deionized water at concentrations of 500 (0.05 wt. %), 1000 (0.1 wt. %), 2000 (0.2 wt. %) and 4000 (0.4 wt. %) ppm. After rheology measurements, zero-shear viscosities of 1.26, 1.59, 2.39, and 8.23 mPa·s, respectively, were obtained.

Fluorescent polystyrene particles (Fluoro-Max, Thermo Fisher Scientific) with diameters of 3 µm (red), 5 µm (green), 7 µm (red) and 10 µm (green) were suspended in the prepared non-Newtonian fluids (with 0.1 % Tween-20 to prevent agglomeration).

### Rheometry analysis

Rheometric analysis was performed using a dual-motor Anton Paar Modular-Compact Rheometer (MCR) 702e Space. Given the low viscosity of the fluid samples, a concentric cylinder geometry (bob-and-cup) was chosen for the measurements, the internal diameter of the exterior cylinder was 45 mm and the diameter of the internal cylinder was 43 mm. Thus, a gap of 1 mm was achieved between the concentric surfaces. The interior cylinder was positioned with a fixed 3 mm gap from the bottom of the exterior cylinder, and for the sake of symmetry, the sample volume was kept at ~ 16 mL to duplicate the 3 mm gap on the top of the interior cylinder. The measurements were performed at ambient temperature (~ 20 °C), similar to the temperature used during the flow experiments. An evaporation preventing lid was placed on the geometries to ensure a constant volume of the fluid samples during the experiments.

The flow curves were obtained through a logarithmic ramp-down on the shear rate starting at 10^3^ 1/s to remove any, if present, memory effects in the sample through a pre-shearing scheme. Depending on the viscosity range of the samples, the lower limits for the shear rate varied from 10 – 10^2^ 1/s for the highest to lowest viscosity fluids. The flow curves showed the viscosity and shear stress as functions of the shear rate in the mentioned range (see Supplementary Material Fig. S7). There were seven (7) points measured per decade of the shear rate, which was kept constant at each point until a steady state was reached. The rheological properties of the four PEO solutions are shown in Supplementary Table S1 (see the Supplementary Material on how relaxation times are calculated; see Tables S2, S3 for the dimensionless numbers).

### Experimental setup and analysis

The microfluidic experiments were performed with a 5 mL steel syringe using a mid-pressure pump (neMESYS CETONI GmbH). The flow rates ranged from 1 – 250 µL/min, and each data point was recorded 5 min after changing the flow rate to ensure the stabilization of flow due to the viscoelastic effect. Imaging was performed using an inverted microscope (Nikon Eclipse TI) with an sCMOS camera (Andor Zyla) and an LED lighting system (Lumenor Spectra X LED). Optical TRITC and FITC filters were used to capture the red and green fluorescent particles, respectively. To control the microscope and record the images, the open-source software Micro Manager was used.

The recorded data were visualized and processed by ImageJ software. The focusing qualities of the particles were calculated based on the ratio of the particle diameter to the full width at half maximum (FWHM) of the fluorescence intensity of the particles along the channel width^61^.

### Numerical approach

We perform three-dimensional direct numerical simulations to investigate the cross-streamline migration of the particles suspended in viscoelastic fluids within a high aspect ratio straight microchannel. These simulations aim to provide additional insights to elucidate the experimental observations. We employ our in-house code^62^ utilizing a direct forcing immersed boundary method (IBM)^63^ to simulate the particles as moving Lagrangian grids. The carrier fluid is discretized within a stationary Eulerian frame, in which the Navier–Stokes and viscoelastic constitutive equations are discretized using finite differences (see the Supplementary Material on code validation). The solver was previously used for particle migration in elasto-viscoplastic channel flow^53^. The suspending fluid motion is governed by the continuity constraint and conservation of momentum as follows:

4 $$\nabla \,\cdot\, U=0,$$

5 $$\left(\frac{\partial U}{\partial t}+\left(U\,\cdot\, \nabla \right){\rm{U}}\right)=-\nabla {\mathsf{p}}{\mathsf{+}}\frac{1}{\mathrm{Re}}\nabla \,\cdot\, \tau +f,$$

where U is the fluid velocity, p is the pressure field, τ is the total deviatoric stress tensor, and Re is the Reynolds number. The extra term f on the right-hand side of Eq. (5) is the immersed boundary force field representing the particle–fluid interaction. Details of the immersed boundary method are given in the Supplementary Material. The total deviatoric stress tensor, *τ*, is composed of contributions from the solvent (Newtonian fluid) and polymer parts as *τ* = *τ*^*s*^ + *τ*^*p*^. The solvent stress tensor is defined as ${\tau }^{s}={\beta }_{s}\left(\nabla U+\nabla {U}^{T}\right)$, where *β*_*s*_ = *μ*_*s*_/*μ* is the ratio of the solvent viscosity to the total viscosity. In addition to the equations mentioned earlier, a constitutive equation needs to be employed to model the evolution of the non-Newtonian contribution (*τ*^*p*^) of the viscoelastic material. We simulate the Oldroyd-B model^64^ to consider the viscoelasticity of the material.

The particle translational and rotational velocities are obtained by solving the Newton–Euler equations for each particle.

6 $${\rho }_{p}{V}_{p}\frac{d{u}_{p}}{{dt}}={\oint }_{\partial V}\sigma \,\cdot\, {n\; dA}+{F}_{c},$$

7 $${I}_{p}\frac{d{\omega }_{p}}{{dt}}={\oint }_{\partial V}r\times \left(\sigma \,\cdot\, n\right){dA}+{T}_{c},$$

where u_*p*_ and ω_*p*_ are the linear velocities of the center of mass and angular velocity of the particle, respectively. Additionally, *σ* denotes the Cauchy stress tensor for the viscoelastic fluid and is defined as *σ* = −*p*I + τ^*p*^ + *βs* (∇*U* + ∇*U*^*T*^), where *r* is the distance from the center of the particle, and *∂V* represents the particle domain. The density, volume, and moment of inertia of the particle are denoted by *ρ*_*p*_, *V*_*p*_, and *I*_*p*_, respectively. Finally, *F*_*c*_ and *T*_*c*_ represent the total force and torque generated by potential particle‒wall collisions, respectively.

### Supplementary information

Updated Supplementary information

## References

[1] Sackmann EK, Fulton AL, Beebe DJ (2014). The present and future role of microfluidics in biomedical research. Nature.

[2] Zhu X (2022). Microfluidics as an emerging platform for exploring soil environmental processes: a critical review. Environ. Sci. Technol..

[3] Bhagat AAS (2010). Microfluidics for cell separation. Med. Biol. Eng. Comput..

[4] Sun B, Jiang J, Shi N, Xu W (2016). Application of microfluidics technology in chemical engineering for enhanced safety. Process Saf. Prog..

[5] Whitesides GM (2006). The origins and the future of microfluidics. Nature.

[6] Di Carlo D, Irimia D, Tompkins RG, Toner M (2007). Continuous inertial focusing, ordering, and separation of particles in microchannels. Proc. Natl. Acad. Sci. USA.

[7] Kralj JG, Lis MTW, Schmidt MA, Jensen KF (2006). Continuous dielectrophoretic size-based particle sorting. Anal. Chem..

[8] Pamme N, Manz A (2004). On-chip free-flow magnetophoresis: continuous flow separation of magnetic particles and agglomerates. Anal. Chem..

[9] Petersson F, Åberg L, Swärd-Nilsson A-M, Laurell T (2007). Free flow acoustophoresis: microfluidic-based mode of particle and cell separation. Anal. Chem..

[10] Narayanamurthy V (2020). Advances in passively driven microfluidics and lab-on-chip devices: a comprehensive literature review and patent analysis. RSC Adv..

[11] Zhang S, Wang Y, Onck P, Den Toonder J (2020). A concise review of microfluidic particle manipulation methods. Microfluid. Nanofluidics.

[12] Yamada M, Nakashima M, Seki M (2004). Pinched flow fractionation: continuous size separation of particles utilizing a laminar flow profile in a pinched microchannel. Anal. Chem..

[13] Martel JM, Toner M (2014). Inertial focusing in microfluidics. Annu. Rev. Biomed. Eng..

[14] Wu Z, Willing B, Bjerketorp J, Jansson JK, Hjort K (2009). Soft inertial microfluidics for high throughput separation of bacteria from human blood cells. Lab. Chip..

[15] Zhu Z (2022). High-throughput and label-free enrichment of malignant tumor cells and clusters from pleural and peritoneal effusions using inertial microfluidics. Lab. Chip.

[16] Tavassoli H (2021). Label-free isolation and single cell biophysical phenotyping analysis of primary cardiomyocytes using inertial microfluidics. Small.

[17] Segré G, Silberberg A (1961). Radial particle displacements in poiseuille flow of suspensions. Nature.

[18] Mashhadian A, Shamloo A (2019). Inertial microfluidics: a method for fast prediction of focusing pattern of particles in the cross section of the channel. Anal. Chim. Acta..

[19] Di Carlo D, Edd JF, Humphry KJ, Stone HA, Toner M (2009). Particle segregation and dynamics in confined flows. Phys. Rev. Lett..

[20] Mukherjee P, Wang X, Zhou J, Papautsky I (2019). Single stream inertial focusing in low aspect-ratio triangular microchannels. Lab. Chip.

[21] Liu C, Hu G, Jiang X, Sun J (2015). Inertial focusing of spherical particles in rectangular microchannels over a wide range of Reynolds numbers. Lab. Chip.

[22] Bhagat AAS, Kuntaegowdanahalli SS, Papautsky I (2008). Enhanced particle filtration in straight microchannels using shear-modulated inertial migration. Phys. Fluids.

[23] Cruz J, Hjort K (2021). High-resolution particle separation by inertial focusing in high aspect ratio curved microfluidics. Sci. Rep..

[24] Zhou J, Papautsky I (2020). Viscoelastic microfluidics: progress and challenges. Microsyst. Nanoeng..

[25] Seo KW, Kang YJ, Lee SJ (2014). Lateral migration and focusing of microspheres in a microchannel flow of viscoelastic fluids. Phys. Fluids.

[26] Charjouei Moghadam M, Eilaghi A, Rezai P (2021). Elasto-inertial microparticle focusing in straight microchannels: A numerical parametric investigation. Phys. Fluids.

[27] Villone MM, D’Avino G, Hulsen MA, Greco F, Maffettone PL (2013). Particle motion in square channel flow of a viscoelastic liquid: Migration vs. secondary flows. J. Non-Newton. Fluid Mech..

[28] Raoufi MA (2019). Experimental and numerical study of elasto-inertial focusing in straight channels. Biomicrofluidics.

[29] Liu C, Xue C, Hu G (2015). Sheathless separation of particles and cells by viscoelastic effects in straight rectangular microchannels. Procedia Eng..

[30] Yang S, Kim JY, Lee SJ, Lee SS, Kim JM (2011). Sheathless elasto-inertial particle focusing and continuous separation in a straight rectangular microchannel. Lab. Chip..

[31] Etcheverry S (2017). High performance micro-flow cytometer based on optical fibres. Sci. Rep..

[32] Seo KW, Byeon HJ, Huh HK, Lee SJ (2014). Particle migration and single-line particle focusing in microscale pipe flow of viscoelastic fluids. RSC Adv.

[33] Kumar T (2021). High throughput viscoelastic particle focusing and separation in spiral microchannels. Sci. Rep..

[34] Bai J-J (2023). Dean-flow-coupled elasto-inertial focusing accelerates exosome purification to facilitate single vesicle profiling. Anal. Chem..

[35] Nam J, Lim H, Kim D, Jung H, Shin S (2012). Continuous separation of microparticles in a microfluidic channel via the elasto-inertial effect of non-newtonian fluid. Lab. Chip.

[36] Faridi MA (2017). Elasto-inertial microfluidics for bacteria separation from whole blood for sepsis diagnostics. J. Nanobiotechnology.

[37] Lu X, Xuan X (2015). Continuous microfluidic particle separation via elasto-inertial pinched flow fractionation. Anal. Chem..

[38] Squires TM, Quake SR (2005). Microfluidics: fluid physics at the nanoliter scale. Rev. Mod. Phys..

[39] Di Carlo D (2009). Inertial microfluidics. Lab. Chip..

[40] Wlodkowic D (2010). Microfluidics: emerging prospects for anti-cancer drug screening. World J. Clin. Oncol..

[41] Zhou J, Papautsky I (2013). Fundamentals of inertial focusing in microchannels. Lab. Chip.

[42] Bretherton FP (1962). The motion of rigid particles in a shear flow at low reynolds number. J. Fluid Mech..

[43] Hu X, Lin J, Chen D, Ku X (2020). Stability condition of self-organizing staggered particle trains in channel flow. Microfluid. Nanofluidics.

[44] Li D, Xuan X (2019). The motion of rigid particles in the poiseuille flow of pseudoplastic fluids through straight rectangular microchannels. Microfluid. Nanofluidics.

[45] D’Avino G, Maffettone PL (2015). Particle dynamics in viscoelastic liquids. J. Non-Newton. Fluid Mech..

[46] Yang SH, Lee DJ, Youn JR, Song YS (2017). Multiple-line particle focusing under viscoelastic flow in a microfluidic device. Anal. Chem..

[47] Zhou Y, Ma Z, Ai Y (2020). Dynamically tunable elasto-inertial particle focusing and sorting in microfluidics. Lab. Chip.

[48] Li G, McKinley GH, Ardekani AM (2015). Dynamics of particle migration in channel flow of viscoelastic fluids. J. Fluid Mech..

[49] Lu X, Zhu L, Hua R, Xuan X (2015). Continuous sheath-free separation of particles by shape in viscoelastic fluids. Appl. Phys. Lett..

[50] Li D, Lu X, Xuan X (2016). Viscoelastic separation of particles by size in straight rectangular microchannels: a parametric study for a refined understanding. Anal. Chem..

[51] Naderi MM, Barilla L, Zhou J, Papautsky I, Peng Z (2022). Elasto-inertial focusing mechanisms of particles in shear-thinning viscoelastic fluid in rectangular microchannels. Micromachines.

[52] Demiral D, Boes RM, Albayrak I (2020). Effects of secondary currents on turbulence characteristics of supercritical open channel flows at low aspect ratios. Water.

[53] Chaparian E, Ardekani MN, Brandt L, Tammisola O (2020). Particle migration in channel flow of an elastoviscoplastic fluid. J. Non-Newton. Fluid Mech..

[54] Liu C (2015). Size-based separation of articles and cells utilizing viscoelastic effects in straight microchannels. Anal. Chem..

[55] Liu C (2017). Field-free isolation of exosomes from extracellular vesicles by microfluidic viscoelastic flows. ACS Nano.

[56] Tian F (2018). Label-free isolation of rare tumor cells from untreated whole blood by interfacial viscoelastic microfluidics. Lab. Chip..

[57] Ramachandraiah H (2014). Dean flow-coupled inertial focusing in curved channels. Biomicrofluidics.

[58] Feng H (2022). Viscoelastic particle focusing and separation in a spiral channel. Micromachines.

[59] Narayana Iyengar S, Kumar T, Mårtensson G, Russom A (2021). High resolution and rapid separation of bacteria from blood using elasto-inertial microfluidics. Electrophoresis.

[60] Johnson, D. W., Goettert, J., Singh, V. & Yemane, D. *SUEX Dry Film Resist—A new Material for High Aspect Ratio Lithography*. https://www.lsu.edu/camd/files/DJ_AR2012_SUEXoverview.pdf (2012).

[61] Gao H, Zhou J, Naderi MM, Peng Z, Papautsky I (2023). Evolution of focused streams for viscoelastic flow in spiral microchannels. Microsyst. Nanoeng..

[62] Izbassarov D (2018). Computational modeling of multiphase viscoelastic and elastoviscoplastic flows. Int. J. Numer. Methods Fluids.

[63] Breugem W-P (2012). A second-order accurate immersed boundary method for fully resolved simulations of particle-laden flows. J. Comput. Phys..

[64] Oldroyd JG, Wilson AH (1950). On the formulation of rheological equations of state. Proc. R. Soc. Lond. Ser. Math. Phys. Sci..

