# Supplementary material for: Elasto-inertial focusing and particle migration in high aspect ratio microchannels for high-throughput separation
Source: Microsyst Nanoeng. 2024 Jun 25;10:87. doi: 10.1038/s41378-024-00724-2 (PMC11196675; doi:10.1038/s41378-024-00724-2)
Supplement: Supplementary file 1 — Updated Supplementary information [file 41378_2024_724_MOESM1_ESM.pdf]

Elasto-inertial focusing and particle migration in high aspect ratio microchannels for high-throughput applications

Supplementary Information

Selim Tanriverdi [1], Javier Cruz[1,2], Shahriar Habibi[3], Kasra Amini[4], Martim Costa[1], Fredrik Lundell[4,5], Gustaf Mårtensson[1], Luca Brandt[3,6], Outi Tammisola[3] & Aman Russom [1,7,*]

1. *Division of Nanobiotechnology, Department of Protein Science, Science for Life Laboratory, KTH Royal Institute of Technology, 171 65, Solna, Sweden. E-mail: aman@kth.se*
2. *Division of Microsystems Technology, Department of Materials Science and Engineering, Uppsala University, 752 37, Uppsala, Sweden*
3. *FLOW and SeRC (Swedish e-Science Research Centre), Department of Engineering Mechanics, Royal Institute of Technology, SE 100 44, Stockholm, Sweden*
4. *FLOW and Fluid Physics Laboratory, Department of Engineering Mechanics, Royal Institute of Technology, Stockholm, Sweden*
5. *Wallenberg Wood Science Center, Royal Institute of Technology, SE 100 44, Stockholm, Sweden*
6. *Department of Energy and Process Engineering, Norwegian University of Science and Technology (NTNU), Trondheim, Norway*
7. *AIMES-Center for the Advancement of Integrated Medical and Engineering Sciences at Karolinska Institutet and KTH Royal Institute of Technology, Stockholm, Sweden*

Effect of particle size

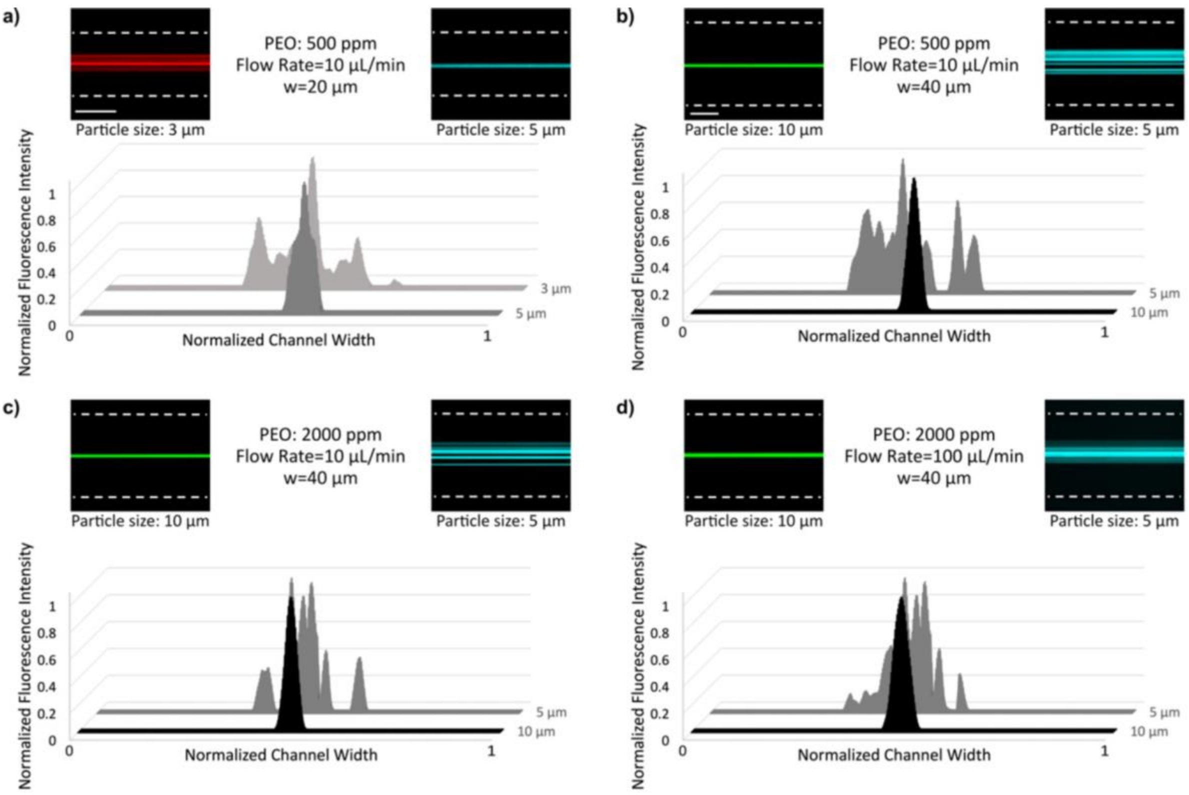

Fig. S1 Effect of particle size on focusing for various conditions. a 3-5 µm particles in microchannel (*h*=60 µm, *w*=20 µm) at 10 µL/min and 500 ppm of PEO **b** 5-10 µm particles in microchannel (*h*=60 µm, *w*=40 µm) at 10 µL/min and 500 ppm of PEO **c** 5-10 µm particles in microchannel (*h*=60 µm, *w*=40 µm) at 10 µL/min and 2000 ppm of PEO **d** 5-10 µm particles in microchannel (*h*=60 µm, *w*=40 µm) at 100 µL/min and 2000 ppm of PEO. Scale bars:100 µm

Particles in lower stress region

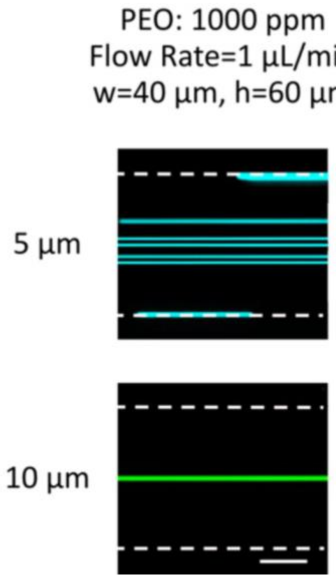

Fig. S2 Particles in the corner. 5 µm and 10 µm particles flowing through the channel, where some of the 5 µm particles are trapped in the corner of the channel while 10 µm particles are focused in the channel center under the same experimental conditions. Scale bar: 100 µm

Effect of channel height

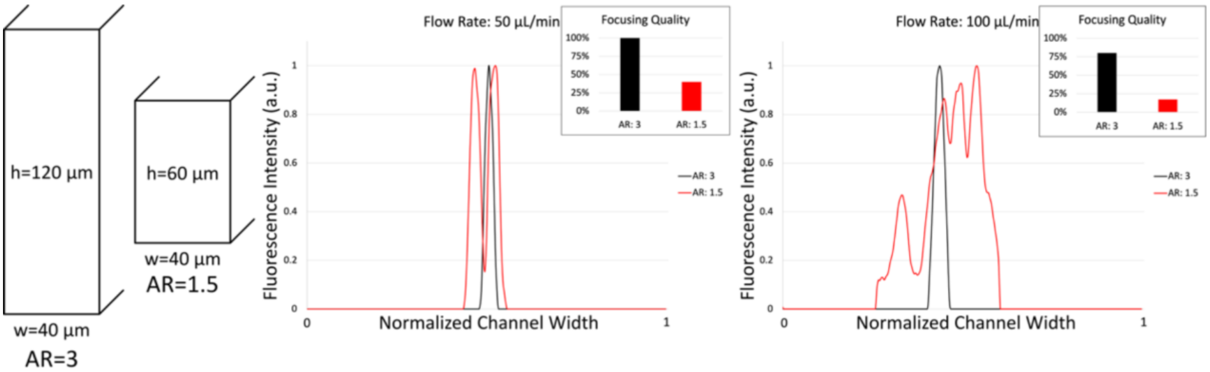

Fig. S3 Effect of channel height. Fluorescence intensity graph and focusing quality of 10 µm particles in microchannels (h=60, 120 µm, w=40 µm). At flow rate 50 µL/min (1000 ppm) and at 100 µL/min (500 ppm)

Effect of channel length

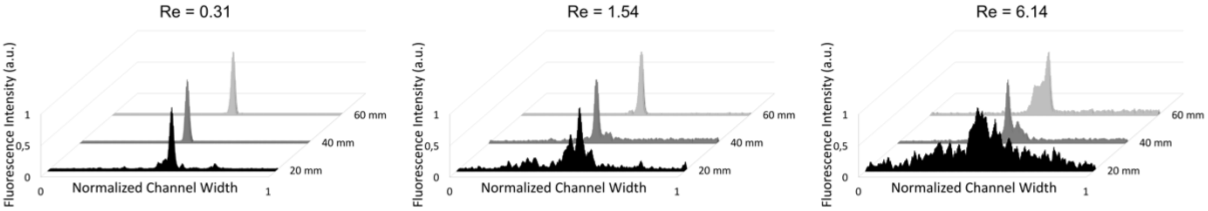

Fig. S4 Effect of channel length. Fluorescence intensity graph of 1 µm particles in microchannels (h=15 µm, w=20 µm) at three different lengths (20, 40, 60 mm) in 1000 ppm PEO solution. At flow rate 0.5 µL/min (Re: 0.31), 2.5 µL/min (Re:1.54) and 10 µL/min (Re: 6.14)

Effect of PEO concentration

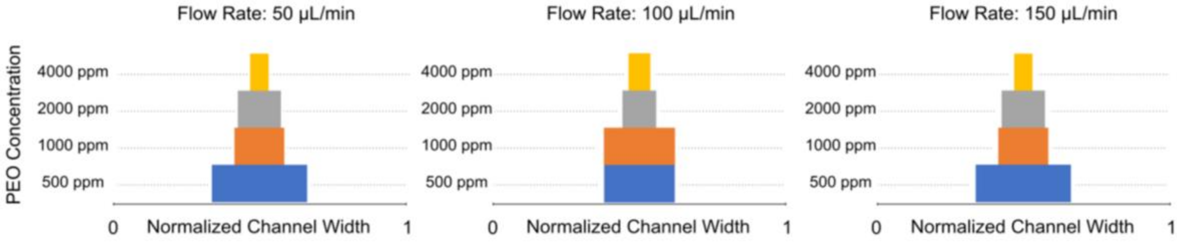

Fig. S5 Effect of PEO concentration on focusing of 5 µm particles.

Analysis of final particle position at outlet

Elasto-inertial particle focusing is complex and the relationship between the forces affecting the particle migration towards the equilibrium position is not well studied. In an effort to understand the trends, we examined the final particle lateral position at the outlets (position 9). The outcome is summarized in Fig. S6. To analyze the final particle positions at the outlet, we denominated W_n as the normalized channel width, from 0 at the inner wall to 1 at the outer wall of the channel. Moreover, for easy of analysis, we divided the channel cross section into 5 separate regions. Region 0 begins at the inner wall, where the particles are located after the channel split, and ends at $0.2W_n$. Region 1 extends from 0.2 to $0.27W_n$, Region 2 from 0.28 to $0.35W_n$, Region 3 from 0.36 to $0.43W_n$ and Region 4 from 0.44 to $0.52W_n$ (see Fig. S6a). The channel center corresponds to $0.5W_n$. After the channel split (at position 1, see Fig. 1), focused

particles are close to the channel wall in Region 0, and start to migrate toward the channel center (Region 4). Particles are imaged at the outlet of the channel (position 9) for different flow rates and PEO concentrations.

The analysis of particle position at the channel outlet based on fluorescence intensity is summarized in Fig. S6. The collection of all experimental conditions, which we analyzed the position of particles is shown in Fig. S6a. The results show that particles are most likely to be found at the channel center (Region 4) as expected and the second most probable location for particles is noticed at the region 0.28-0.35W_n (Region 2).

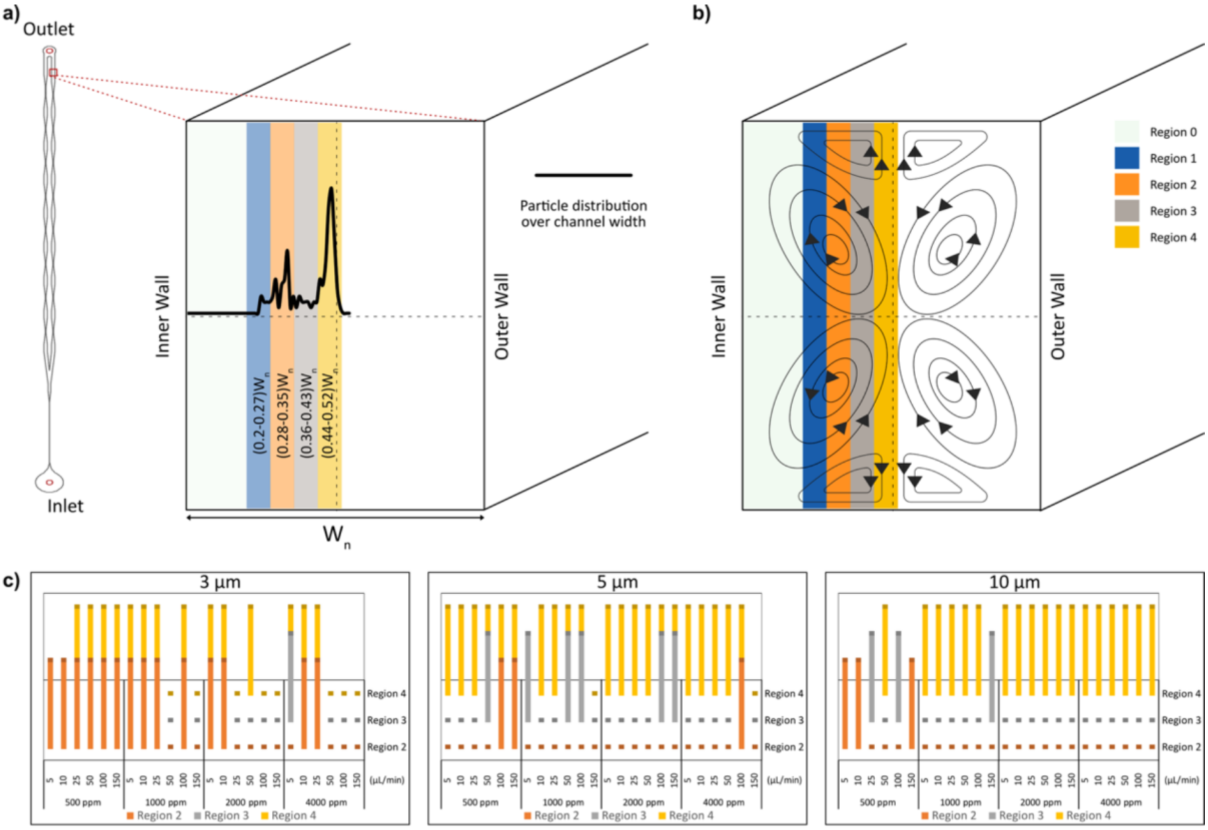

Fig. S6 Lateral particle focusing positions at the channel outlet (position 9). a Cross-sectional intensity summerizing all the experimental conditions for 3-5-10 µm particles. **b** Schematic of secondary flow streamlines in high aspect ratio microchannels. **c** Final position of 3-5-10 µm particles at different PEO concentrations (from 500 ppm to 4000 ppm) and flow rates (5, 10, 25, 50, 100 and 150 µL/min).

We found that an increase of the flow rate causes stronger inertial and elastic forces, and thus enhances the secondary cross-flow induced by the second normal stress difference, N_2.[1] Based on our results shown in Fig. S6a, we show the secondary flow streamlines for the high aspect ratio microchannels at position 9 as illustrated in Fig. S6b. The center of these streamlines corresponds to the Region 2 where we have observed a second equilibrium position, in addition to midplane, Region 4 (see Fig. S6a). These observations suggest a role of the N_2-induced secondary flow at high flow rates when inertia becomes important. Here, we also believe that Region 3, where flow streamlines are assumed to be stronger, acts as a barrier. If the forces that move the particles overcome this barrier, particles are likely to be in the Region 4, channel center. As the forces strongly depend on the particle sizes, we observe that larger particles are able to pass this barrier and focus in the Region 4 easier than smaller particles. Similar results are found for different PEO concentrations: the majority of the particles are

found in Region 2 or 4. All three different particle sizes are observed in Region 2 the highest, when the PEO concentration is lowest (500 ppm). Increasing the PEO concentration to 4000 ppm reduces the number of particles found in Region 2 and increases the number of particles found in Region 4 for all particle sizes (see Fig. S6c). Moreover, this change is more significant for the largest particle (10 µm), inducing all 10 µm particles to be focused in Region 4 at PEO concentration of 2000 and 4000 ppm (see Fig. S6c).

Rheometry analysis

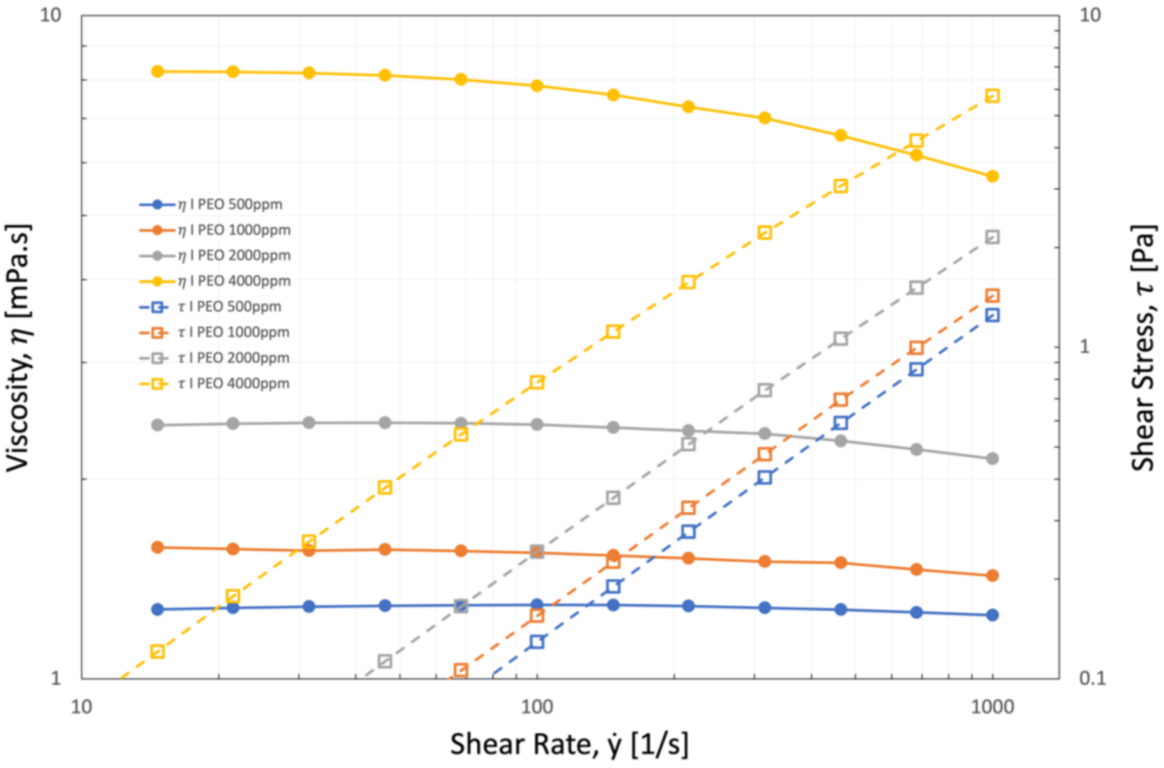

Fig. S7 Shear viscosities of solutions at different PEO concentrations.

	PEO Concentration (ppm)			
	**500**	**1000**	**2000**	**4000**
**Density (kg/m³)**	996	996	996	996
**Zero-shear viscosity μ_0 (mPa.s)**	1.26	1.59	2.39	8.23
**Effective relaxation time λe (ms)**	4.31	6.76	10.61	16.65
**Overlap concentration c* (ppm)**	858	858	858	858

Table S1 Rheological properties of solutions containing PEO of $M_W=2\times10^6$ g/mol

Calculation of relaxation time

The relaxation times for different concentrations of PEO were estimated from the following formulate that was suggested by Tirtaatmadja et al[2]

$$\lambda_e = 18\lambda_{Zimm}(c/c^*)^{0.65}$$

where λ_{Zimm} is the relaxation time that is predicted from Zimm Theory[2] and c* is the polymer overlap concentration.

$$\lambda_{Zimm} = F\ \frac{[\eta] M_W \eta_s}{N_A k_B T}$$

where [η] is the intrinsic viscosity, η_s is the solvent viscosity (water, η_s = 1 mPa.s), N_A is the Avogadro's constant, k_B is Boltzmann's constant and T is the absolute temperature. The front factor F is estimated by the Riemann Zeta function[2] and found 0.463. PEO solution in our experiments has the M_w of 2x10^6 g/mol. Intrinsic viscosity of PEO is calculated using Mark-Houwink-Sakurada equation[3];

$$[\eta] = 0.072 M_W^{0.65} = 897\ ml/g$$

Overlap concentration of PEO is calculated using the Graessley equation[4];

$$c^* = 0.77/[\eta] = 858\ ppm$$

By putting all the parameters in Zimm theory, we obtain λ_{Zimm} as 0.34 ms and we calculate the effective relaxation times for four different concentrations of PEO solutions and the dimensionless numbers Wi, El as shown in Table S2 and S3.

Q (µL/min)	PEO Concentration											
	500 ppm			1000 ppm			2000 ppm			4000 ppm		
	Re	Wi	El	Re	Wi	El	Re	Wi	El	Re	Wi	El
1	0.27	1.55	5.76	0.21	2.43	11.40	0.14	3.81	26.89	0.04	5.98	145.11
2.5	0.67	3.87	5.76	0.53	6.07	11.40	0.35	9.53	26.89	0.10	14.95	145.11
5	1.34	7.74	5.76	1.07	12.14	11.40	0.71	19.06	26.89	0.21	29.90	145.11
10	2.69	15.48	5.76	2.13	24.28	11.40	1.42	38.11	26.89	0.41	59.81	145.11
25	6.72	38.70	5.76	5.33	60.70	11.40	3.54	95.28	26.89	1.03	149.52	145.11
50	13.44	77.41	5.76	10.65	121.41	11.40	7.09	190.55	26.89	2.06	299.03	145.11
100	26.89	154.81	5.76	21.31	242.82	11.40	14.17	381.11	26.89	4.12	598.06	145.11
150	40.33	232.22	5.76	31.96	364.22	11.40	21.26	571.66	26.89	6.18	897.09	145.11
250	67.22	387.03	5.76	53.27	607.04	11.40	35.44	952.77	26.89	10.30	1495.15	145.11

Table S2 Dimensionless numbers in microchannel with height=60 µm and width=40 µm

Q (µL/min)	PEO Concentration											
	500 ppm			1000 ppm			2000 ppm			4000 ppm		
	Re	Wi	El	Re	Wi	El	Re	Wi	El	Re	Wi	El
1	0.34	6.19	18.33	0.27	9.71	36.28	0.18	15.24	85.60	0.05	23.92	461.99
2.5	0.84	15.48	18.33	0.67	24.28	36.28	0.45	38.11	85.60	0.13	59.81	461.99
5	1.69	30.96	18.33	1.34	48.56	36.28	0.89	76.22	85.60	0.26	119.61	461.99
10	3.38	61.93	18.33	2.68	97.13	36.28	1.78	152.44	85.60	0.52	239.22	461.99
25	8.45	154.81	18.33	6.69	242.82	36.28	4.45	381.11	85.60	1.29	598.06	461.99
50	16.89	309.63	18.33	13.38	485.63	36.28	8.90	762.21	85.60	2.59	1196.12	461.99
100	33.78	619.25	18.33	26.77	971.26	36.28	17.81	1524.43	85.60	5.18	2392.24	461.99
150	50.67	928.88	18.33	40.15	1456.90	36.28	26.71	2286.64	85.60	NA	NA	NA
250	84.45	1548.13	18.33	66.92	2428.16	36.28	44.52	3811.06	85.60	NA	NA	NA

Table S3 Dimensionless numbers in microchannel with height=60 µm and width=20 µm

Computational domain

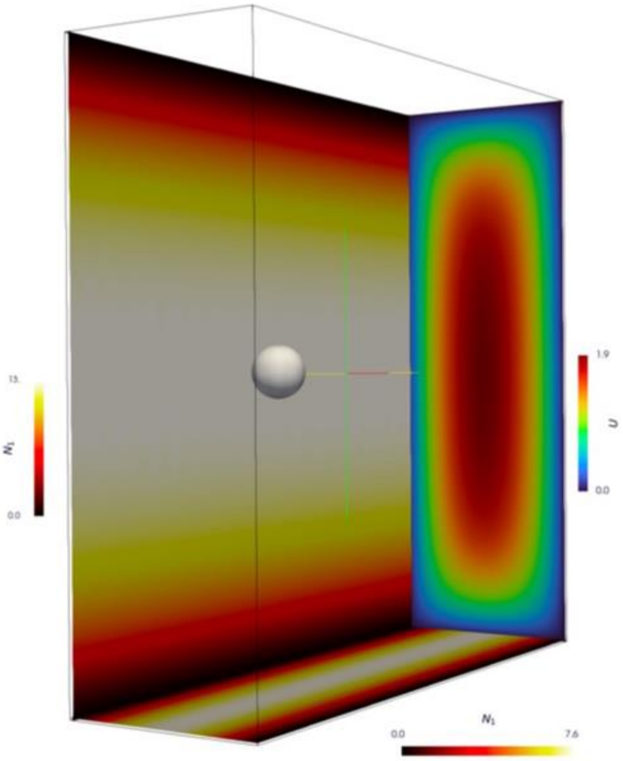

Fig. S8 Three-dimensional view of the computational domain. N_1 and U represent the first normal stress difference and stream-wise velocity in their corresponding planes. The aspect ratio of the channel is 3 and the ratio of the channel height to the particle diameter is 12. The particle has reached an equilibrium position in the middle of the domain.

Fig. S8 presents an illustration of our computational domain, featuring a channel with dimensions of 12D × 12D × 4D, where D denotes the particle diameter. The discretization is achieved through 384 × 384 × 128 Eulerian grid points in the stream-wise, wall-normal, and span-wise directions. The number of Eulerian grid points across the particle diameter is 32 while 3219 Lagrangian grid points are uniformly distributed over the surface of the particles. All the simulations are performed with a constant bulk velocity U_b through the channel to guarantee a fixed Reynolds number. Periodic boundary conditions are imposed for both fluid and particles in the stream-wise direction, whereas the no-slip and no-penetration boundary conditions are applied at the top, bottom, left, and right walls of the domain. The Navier-Stokes equations are solved by means of a projection method (pressure correction) to decouple the computations of the velocity and pressure fields and by implementing a highly efficient and scalable FFT-based method to solve the pressure Poisson equation. In addition, the spatial derivatives in flow equations are approximated by a central finite difference scheme, with the exception of the advection term in the constitutive equation, which is discretized using the fifth-order WENO method.[5] Furthermore, a third-order Runge-Kutta (RK3) scheme is used to integrate the governing equations in time. The fluid-solid interaction is simulated by a direct forcing IBM first proposed by Uhlmann[6] and further improved by Breugem.[7] In the IBM method, the fluid is represented by a uniform (Δx = Δy = Δz) staggered

Cartesian grid and the particle interface is represented by a collection of moving Lagrangian points that are uniformly distributed on the surface of the particle. The Navier-Stokes equations are solved on the entire grid including points within the particles, and the effect of the presence of the particles is modelled by adding an extra force f on the right-hand side of Eq. (5) in the immediate vicinity of the solid boundary to the fluid which enforces the no-slip/no-penetration (ns/np) condition on the surface of the particles.[8] The obtained IB force is applied to both dispersed and carrier phases to update the velocities in time. The reader can find detailed explanations of the numerical approach used in the present work in our previous studies.[9–11]

Code validation

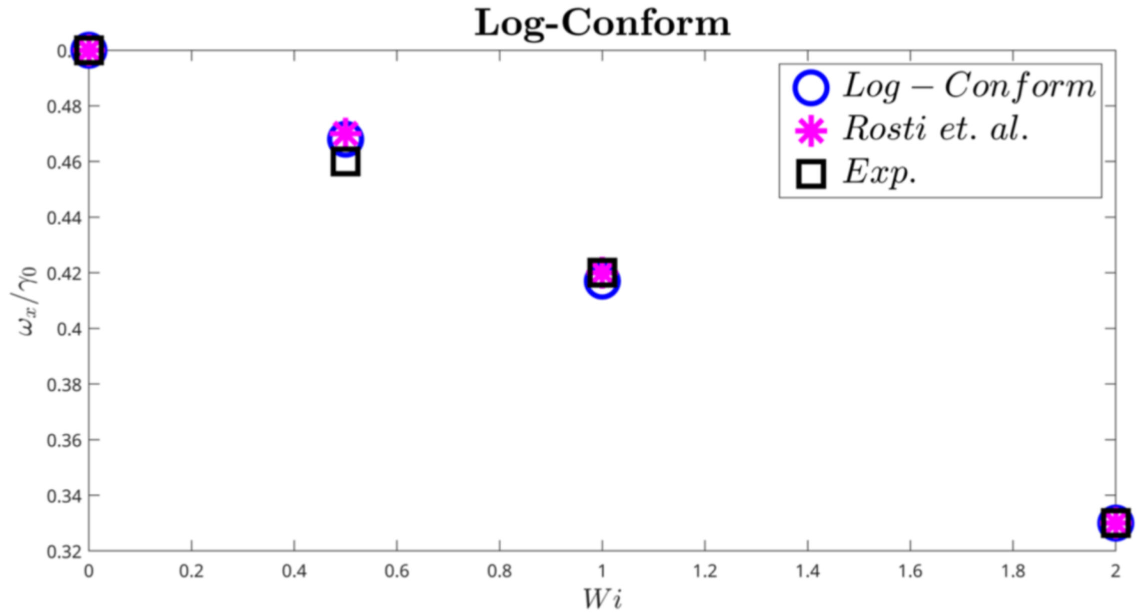

Fig. S9 The angular velocity of the particle relative to the x axis normalized by the shear rate is calculated across different Weissenberg numbers. The blue circles denote the numerical results, the black squares the experiments of Snijkers et al.[12], and the purple symbols are from the numerical code in Goyal & Derksen[13]

The numerical code has been broadly used and validated in our previous publications, for simulating spherical and oblate particles in a Newtonian fluid in microchannels[13], for simulating elastic particles[15,16] in shear flows, and for simulating particles in viscoelastic turbulent flows.[17] The box size and resolution studies were done in a previous study.[14] In this context, we present an additional validation case, focusing on the rotation of a spherical particle in a viscoelastic Couette flow, aiming to reassess the accuracy of our viscoelastic solver and immersed boundary method. Specifically, we consider a spherical particle of radius R positioned in the center of an elastic Couette flow. The computational domain, measuring 4R × 8R × 8R, is discretized with 24 grid points per particle diameter. The top and bottom walls exhibit opposite velocities Vw, generating a shear rate $\gamma = 2Vw/8R$, while periodic boundary conditions are applied in the remaining directions. The particle is neutrally buoyant (the density of the particle and the carrier fluid are the same), and the Reynolds number $Re = \rho\gamma R^2/\mu$ is fixed at 0.025. The fluid is described by the Oldroyd-B equation, and the Weissenberg number $Wi = \lambda\gamma$ varies from 0 to 2. The movement of the planes induces rotation in the particle. The magnitude of this rotation is a function of Re and Wi numbers.

We compute the particle angular velocity with respect to the x axis across various Weissenberg numbers and validate our results with the experiments conducted by Snijkers.[12] Fig. S9 reveals a very good agreement between our numerical simulations and the experiments from Snijkers. This underscores the accuracy of our numerical solver and the effectiveness of the immersed boundary method implemented in our code. It is noticeable that the magnitude of the particle's rotation decreases with an increase in the fluid's elasticity, as evident from the simulations and experiments.

References

1. Speziale, C. G. On turbulent secondary flows in pipes of noncircular cross-section. *Int. J. Eng. Sci.* **20**, 863–872 (1982).
2. Tirtaatmadja, V., McKinley, G. H. & Cooper-White, J. J. Drop formation and breakup of low viscosity elastic fluids: Effects of molecular weight and concentration. *Phys. Fluids* **18**, 043101 (2006).
3. Holzner, G., Stavrakis, S. & deMello, A. Elasto-Inertial Focusing of Mammalian Cells and Bacteria Using Low Molecular, Low Viscosity PEO Solutions. *Anal. Chem.* **89**, 11653–11663 (2017).
4. Graessley, W. W. Entangled linear, branched and network polymer systems — Molecular theories. in *Synthesis and Degradation Rheology and Extrusion* 67–117 (Springer Berlin Heidelberg, Berlin, Heidelberg, 1982).
5. Shu, C.-W. High Order Weighted Essentially Nonoscillatory Schemes for Convection Dominated Problems. *SIAM Rev.* **51**, 82–126 (2009).
6. Uhlmann, M. An immersed boundary method with direct forcing for the simulation of particulate flows. *J. Comput. Phys.* **209**, 448–476 (2005).
7. Breugem, W.-P. A second-order accurate immersed boundary method for fully resolved simulations of particle-laden flows. *J. Comput. Phys.* **231**, 4469–4498 (2012).
8. Mittal, R. & Iaccarino, G. IMMERSED BOUNDARY METHODS. *Annu. Rev. Fluid Mech.* **37**, 239–261 (2005).
9. Ardekani, M. N., Costa, P., Breugem, W. P. & Brandt, L. Numerical study of the sedimentation of spheroidal particles. *Int. J. Multiph. Flow* **87**, 16–34 (2016).
10. Izbassarov, D. *et al.* Computational modeling of multiphase viscoelastic and elastoviscoplastic flows. *Int. J. Numer. Methods Fluids* **88**, 521–543 (2018).
11. Niazi Ardekani, M., Brandt, L. & Iaccarino, G. *Numerical Study of Transport Phenomena in Particle Suspensions*. (KTH Royal Institute of Technology, 2019).
12. Snijkers, F. *et al.* Effect of viscoelasticity on the rotation of a sphere in shear flow. *J. Non-Newton. Fluid Mech.* **166**, 363–372 (2011).
13. Goyal, N. & Derksen, J. J. Direct simulations of spherical particles sedimenting in viscoelastic fluids. *J. Non-Newton. Fluid Mech.* **183–184**, 1–13 (2012).
14. Lashgari, I., Ardekani, M. N., Banerjee, I., Russom, A. & Brandt, L. Inertial migration of spherical and oblate particles in straight ducts. *J. Fluid Mech.* **819**, 540–561 (2017).
15. Rosti, M. E., Brandt, L. & Mitra, D. Rheology of suspensions of viscoelastic spheres: Deformability as an effective volume fraction. *Phys. Rev. Fluids* **3**, 012301 (2018).
16. Rosti, M. E. & Brandt, L. Suspensions of deformable particles in a Couette flow. *J. Non-Newton. Fluid Mech.* **262**, 3–11 (2018).
17. Rosti, M. E. & Brandt, L. Increase of turbulent drag by polymers in particle suspensions. *Phys. Rev. Fluids* **5**, 041301 (2020).
